# Ascorbate Peroxidase and Catalase Activities and Their Genetic Regulation in Plants Subjected to Drought and Salinity Stresses

**DOI:** 10.3390/ijms160613561

**Published:** 2015-06-12

**Authors:** Adriano Sofo, Antonio Scopa, Maria Nuzzaci, Antonella Vitti

**Affiliations:** School of Agricultural, Forestry, Food and Environmental Sciences, University of Basilicata, 85100 Potenza, Italy; E-Mails: adriano.sofo@unibas.it (Ad.S.); antonio.scopa@unibas.it (An.S.); maria.nuzzaci@unibas.it (M.N.)

**Keywords:** abiotic stress, reactive oxygen species (ROS), hydrogen peroxide (H_2_O_2_), catalase (CAT), ascorbate peroxidase (APX)

## Abstract

Hydrogen peroxide (H_2_O_2_), an important relatively stable non-radical reactive oxygen species (ROS) is produced by normal aerobic metabolism in plants. At low concentrations, H_2_O_2_ acts as a signal molecule involved in the regulation of specific biological/physiological processes (photosynthetic functions, cell cycle, growth and development, plant responses to biotic and abiotic stresses). Oxidative stress and eventual cell death in plants can be caused by excess H_2_O_2_ accumulation. Since stress factors provoke enhanced production of H_2_O_2_ in plants, severe damage to biomolecules can be possible due to elevated and non-metabolized cellular H_2_O_2_. Plants are endowed with H_2_O_2_-metabolizing enzymes such as catalases (CAT), ascorbate peroxidases (APX), some peroxiredoxins, glutathione/thioredoxin peroxidases, and glutathione sulfo-transferases. However, the most notably distinguished enzymes are CAT and APX since the former mainly occurs in peroxisomes and does not require a reductant for catalyzing a dismutation reaction. In particular, APX has a higher affinity for H_2_O_2_ and reduces it to H_2_O in chloroplasts, cytosol, mitochondria and peroxisomes, as well as in the apoplastic space, utilizing ascorbate as specific electron donor. Based on recent reports, this review highlights the role of H_2_O_2_ in plants experiencing water deficit and salinity and synthesizes major outcomes of studies on CAT and APX activity and genetic regulation in drought- and salt-stressed plants.

## 1. Introduction

Reactive oxygen species (ROS), that are free radicals and non-radical molecules [1], are key components of the signaling pathways’ network, and act as main regulators of cellular responses and cell physiology of plant to environmental factors [2]. In particular, hydrogen peroxide (H_2_O_2_) is the most important relatively stable non-radical ROS [3], without a net charge [4]. It is produced in the photosynthesizing cells at the level of chloroplasts and peroxisomes, the latter being the major site of intracellular H_2_O_2_ production [5], during the photosynthetic carbon oxidation cycle and, to a lesser extent, during photorespiration, and also in the mitochondria, during the respiratory electron transport chain [2]. Increases in H_2_O_2_ production were reported to occur during a biotic and/or abiotic stress regime, such as pathogen attack, wounding, UV irradiation, exposure to intense light, drought, salinity, and/or chilling [1,3]. As a rule, under normal physiological conditions, ROS, including H_2_O_2_, represent byproducts of many plant metabolic pathways and, therefore, they are continuously synthesized in different cellular compartments [6]. On the other hand, ROS are also scavenged by an antioxidative defense system, whose components are often confined to certain cellular compartments [7].

A feature of ROS is that their accumulation causes an oxidative stress, which is oxidative damage to proteins, DNA, and lipids [8]. Therefore, the equilibrium between ROS production and scavenging is very important [9], taking also into account that the final consequence of an eventual disequilibrium due to adverse environmental factors is the rapid increase of intracellular ROS levels, the so-called “oxidative burst” [1]. In particular, the amount of cell H_2_O_2_, together with other ROS, is a good marker of the extent of oxidative stress. As a consequence, the balance of ascorbate peroxidase (APX), glutathione peroxidase (GPX), and catalase (CAT) activities, representing the main enzymatic H_2_O_2_ scavenging mechanism in plants, is crucial for the suppression of toxic H_2_O_2_ levels in a cell [9]. The enzymes APX, GPX, and CAT are able to scavenge H_2_O_2_ with different mechanisms. Specifically, APX, contrary to CAT, requires an ascorbate and glutathione (GSH) regeneration system, the ascorbate-glutathione cycle. In fact, the first reaction of this cycle, catalyzed by APX, is: H_2_O_2_ + Ascorbate → H_2_O + Monodehydroascorbate (MDA). Like APX, GPX also detoxifies H_2_O_2_ to H_2_O, but it uses GSH directly as a reducing agent. Instead, CAT directly converts H_2_O_2_ into H_2_O and 1/2 O_2_ and, on the contrary of APX, it is more involved in detoxification of H_2_O_2_ than the regulation as a signaling molecule in plants [10]. As reported by Apel and Hirt [9], if the balance of scavenging enzymes changes, compensatory mechanisms are induced (*i.e.*, APX and GPX are up-regulated when CAT activity is reduced in plants).

It was demonstrated that H_2_O_2_ plays an important role as an active signaling molecule leading to different cellular responses [11] depending on the site of its production and also on the interaction between this ROS and specific hormones (*i.e.*, abscisic acid, auxins, ethylene, salicylic acid, nitric oxide) [3]. In fact, H_2_O_2_ can act as signal molecule in regulation of plant growth, morphogenesis and development [3], such as in auxin signaling and gravitropism of maize roots [12], and in somatic embryogenesis stimulation of *Lycium barbarum* [13]. For a long time, H_2_O_2_ has also considered an essential molecule of signal transduction in both abiotic and biotic stresses. Matsuda *et al.* [14] demonstrated that application of H_2_O_2_ at low concentrations induces stress tolerance in plants due to the induction of the synthesis of certain substances similar to other normally synthesized during chilling stress. More recently, it was highlighted that APX1 enzyme has a pivotal role when heat and drought stressors are imposed together, just due to a strong increase of H_2_O_2_ in the cytosol of cell *Arabidopsis*, with a possible cell damage [15]. Among ROS, H_2_O_2_ is the only one able to cross the cellular membrane through the membrane water channels aquaporins and, therefore, to move with water in sites distant from that of its production [16,17]. In addition, it is stable if compared to the other ROS and this is the reason why it is studied as a signal molecule in the regulation of particular biological processes, which comprise those involving tolerance to environmental stresses [1].

Based on these and other recent reports, this review highlights the role of H_2_O_2_ in plants experiencing water deficit and salinity and synthesizes major outcomes of studies on CAT and APX activity and genetic regulation in drought- and salt-stressed plants.

## 2. Catalase (CAT)

### 2.1. Drought and Salinity

**Figure 1:**
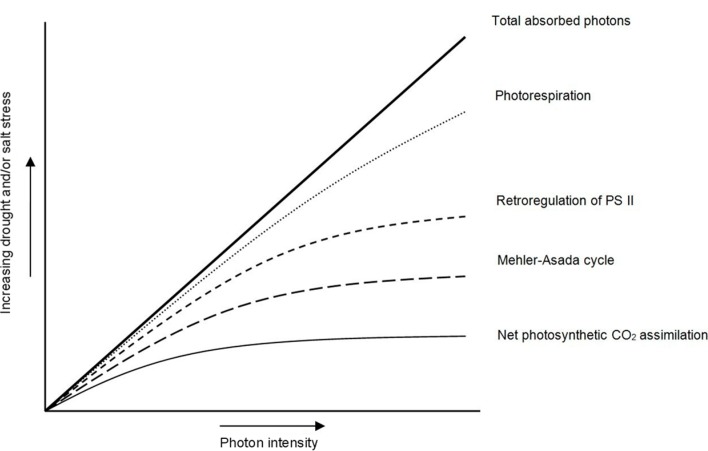
Changes in the destination of absorbed photons with increasing degrees of drought or salinity has effects on some plant physiological and biochemical processes.

Drought stress and salinity are two of the most common abiotic stresses in dry and arid regions. Vegetation dealing with drought stress and salinity has developed a number of physiological mechanisms to grow under adverse climatic conditions [18,19]. Drought stress and salinity are the main causes of reduced plant growth and productivity in semi-arid regions and causes a complex set of responses at molecular, cellular, physiological and developmental levels [20,21]. These responses are mostly due to a photon intensity that exceeds the capacity of stressed plants to absorb it (Figure 1).

It is known that photorespiration makes oxygenic photosynthesis possible by scavenging its major toxic by-product, 2-phosphoglycolate, but also leads to high losses of freshly assimilated CO_2_ from most land plants [22]. Considering the key role of CAT in photorespiration, many authors focused on the role of CAT catalysis pathway under both drought and salt stress. Indeed, the maintenance of CAT activity in leaves of drought-stressed plants likely allowed the removal of photorespiratory H_2_O_2_ produced when plants are subjected to water deficit of salinity, especially under severe degrees of stress. In these conditions, photorespiration works as an energy sink preventing the over-reduction of the photosynthetic electron transport chain and photoinhibition [23]. On this basis, photorespiration and CAT pathway cannot be considered wasteful processes but are nowadays increasingly appreciated as a key ancillary component of photosynthesis and important parts of stress responses in green tissues for preventing ROS accumulation [22,24]. Severe drought stress and salinity predispose the photosynthetic system of leaves to photoinhibition, resulting in a light-dependent inactivation of the primary photochemistry associated with photosystem II, which often persists after rewatering [19]. Indeed, photosynthesis is one of the key processes to be affected by water deficits and high salt contents, via decreased CO_2_ diffusion to the chloroplast and metabolic constraints [20,21]. The relative impact of those limitations varies with the intensity of the stress, the occurrence of superimposed stresses, and the species we are dealing with. Total plant carbon uptake is further reduced due to the concomitant or even earlier inhibition of growth. Leaf carbohydrate status and hormonal ratios are also deeply altered directly by water deficits or indirectly via decreased growth. Acclimation of plants to drought and salinity is often associated with increased levels of reactive oxygen species (ROS), such as superoxide anion (O_2_^−^), hydrogen peroxide (H_2_O_2_), hydroxyl radical (HO^.^) and singlet oxygen (^1^O_2_), which are toxic for the cells [18]. ROS are by-products of aerobic metabolism and their production is enhanced during stress conditions through the disruption of electron transport system, and oxidizing metabolic activities occurring in chloroplasts, mitochondria and microbodies [20].

**Figure 2:**
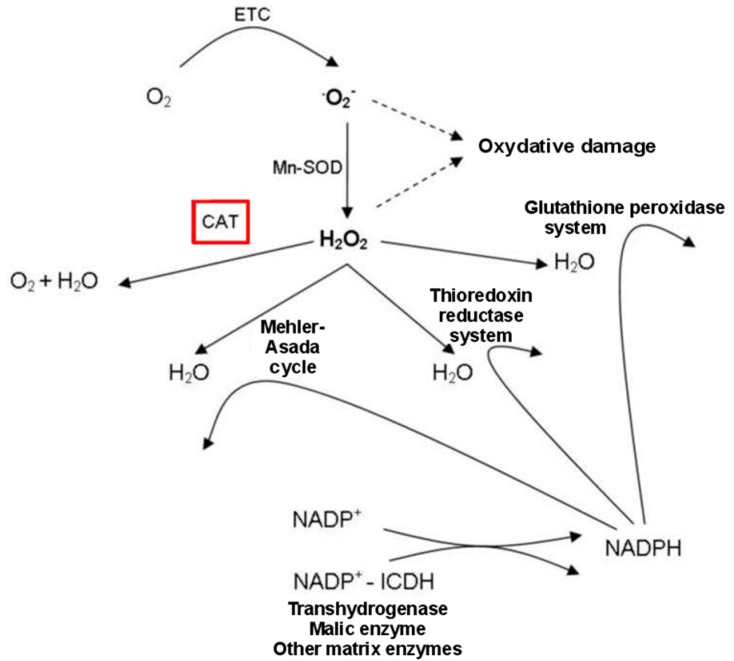
Scavenging of reactive oxygen species (ROS) in plants subjected to abiotic stresses, showing the key role of CAT in ROS scavenging patterns. ETC = electron transport system; ICDH = isocitrate dehydrogenase.

Under non-stressful conditions, ROS are efficiently eliminated by non-enzymatic and enzymatic antioxidants, whereas during drought and saline conditions, the production of ROS exceeds the capacity of the antioxidative systems to remove them, causing oxidative stress [25,26]. In this context, catalase (CAT) isoforms are iron porphyrin enzymes that serve as an efficient ROS scavenging system to avoid the oxidative damage induced to these two stressors [27] (Figure 2).

### 2.2. Drought

An analysis of the recent literature pointed out that an increase in CAT activity is generally positively related to the degree of drought experienced by plants [20,27,28].

In *Panicum sumatrense* under drought stress, the root length increased gradually at all growth stages, while the chlorophyll pigments and plant height showed a reduction [26]. The authors observed that compatible solutes like proline, glycine betaine and free amino acid increased in all drought treatment. Furthermore, stress treatment caused an increase in activity of antioxidant enzymes like superoxide dismutase (SOD), CAT and peroxidases that allow this species to present a high degree of drought tolerance characters. In another drought-tolerant species (*Jatropha curcas*), leaf CO_2_ assimilation rate and carboxylation efficiency parameters decreased progressively as the water deficit increased. In this species, leaf H_2_O_2_ content and lipid peroxidation were inversely and highly correlated with CAT activity, indicating that drought-induced inhibition of this enzyme might have allowed oxidative damage.

Differences between antioxidant responses to drought in C_3_ and C_4_ plants are rather scanty and could be important for understanding the different metabolic antioxidant patterns in these two groups of plants. On this basis, Uzilday *et al.* [29] studied relative shoot growth rate, relative water content and osmotic potential, H_2_O_2_ content and nicotinamide adenine dinucleotide phosphate (NADPH) oxidase activity, CAT activity, *CAT1* mRNA level, and lipid peroxidation in seedlings of *Cleome spinosa* (C_3_) and *Cleome gynandra* (C_4_) exposed to drought stress for 5 and 10 days. They observed that constitutive levels of antioxidant enzymes (except SOD) were consistently higher in *C. spinosa* than in *C. gynandra* under control conditions. CAT1 gene expression in *C. spinosa* was correlated with CAT activity but CAT1 gene expression in *C. gynandra* at 10 days did not show this correlation. Drought stress caused an increase in CAT enzyme levels and activity in both species. The results suggested that in *C. spinosa*, antioxidant defence system was insufficient to suppress the increasing ROS production under stress condition. On the other hand, in *C. gynandra*, although its induction was lower as compared to *C. spinosa*, antioxidant system was able to cope with ROS formation under drought stress. From a proteomic point of view and using a series of multiplexed experiments, Ford *et al.* [30] studied the quantitative changes in protein abundance of three Australian bread wheat cultivars (*Triticum aestivum* L.) in response to drought stress. Three cultivars differing in their ability to maintain grain yield during drought, Kukri (drought-intolerant), Excalibur (drought-tolerant), and RAC875 (drought-tolerant), were grown in the glasshouse with cyclic drought treatment that mimicked conditions in the field. The proteome changes in the three cultivars at the different time points of the water deficit period reflected their differing physiological responses to drought. All three cultivars had changes consistent with an increase in oxidative stress metabolism and ROS scavenging capacity seen through increases in CAT and SOD isoforms, as well as ROS avoidance through the decreases in proteins involved in photosynthesis and the Calvin cycle. The same species was investigated in order to study the response of photosynthesis to drought, heat stress and their combination, by using transgenic wheat line [31]. The researcher revealed that all stresses decreased photosynthesis, although their combination increased the negative effects. In particular, it was showed that drought stress decreased the transpiration rate, stomatal conductance and intercellular CO_2_ concentration. On the contrary, heat stress increased these photosynthetic parameters, but decreased the antioxidant enzyme activity, and hence the antioxidative defense system, to a greater extent.

Scientific work on CAT in tree species are very scarce, considering the difficulty of studying biochemical and molecular responses in field, where a plethora of parameters other than drought have a key role. Sofo *et al.* [32,33] found that olive plants are able to up-regulate the enzymatic antioxidant system as plants enter water deficit conditions. This response protects cellular apparatus and limits cellular damage caused by ROS. In fact, CAT activity showed a significant increase in leaves of drought-stressed plants, reaching values of 11.78 ± 0.18 units·mg^−1^ DW (dry weight). The considerable increase in CAT activity observed in leaves can protect chloroplasts, which under stress conditions present sustained electron flows and are the main producers and targets of ROS action [34].

Is it possible helping plants to face drought stress and/or mitigate its deleterious effects? This possibility has been partially studied in the last years, but we are still far from finding compounds or microorganisms able to induce drought tolerance in plants. In the interesting study of Zhang *et al.* [35], the influence of sudden and gradual drought stress and foliar-applied glycinebetaine (GB) on growth, water relations, osmolyte accumulation and antioxidant defence system were investigated in plants of two maize (*Zea mays* L.) cultivars. Exogenous GB application caused a rise in dry matter, relative water content, contents of GB and free proline as well as the activities of SOD, CAT and GPX to various extents in both cultivars under drought stress. A more pronounced effectiveness of GB application was observed in the drought-sensitive cultivar than that in the drought-tolerant one. From this study, it was possible to propose that hardening for drought resistance by gradual drought stress treatment and GB application are effective to make plants robust to thrive under water-deficit conditions. In order to observe the possible regulatory role of selenium (Se) in relation to the changes in ascorbate (AsA) glutathione (GSH) levels and to the activities of antioxidant pathway enzymes, Hasanuzzaman and Fujita [36] used rapeseed (*Brassica napus*) seedlings grown in Petri dishes and subjected them to two levels of drought stress (10% and 20% PEG). The activity of APX was not affected by drought stress, while CAT activity decreased. On the other hand, Se-pretreated seedlings exposed to drought stress evidenced increased activities of APX and CAT, as compared with the drought-stressed plants without Se. The results indicated that the exogenous application of Se increasesd the tolerance of the plants to drought-induced oxidative damage by enhancing their antioxidant defense.

The effectiveness of autochthonous plant growth-promoting rhizobacteria was studied in *Lavandula dentata* and *Salvia officinalis* growing under drought conditions [37]. These bacteria were identified as *Bacillus megaterium* (Bm), *Enterobacter* sp. (E), *Bacillus thuringiensis*, and *Bacillus* spp. Each bacteria showed different strategies to meliorate water limitation and alleviate drought stress in these two species, including CAT up-regulation. Armada *et al.* [37] demonstrated that particular characteristic of plants, such as low shoot/root ratio and high stomatal conductance are important factors controlling the bacterial effectiveness improving nutritional, physiological, and metabolic plant activities.

### 2.3. Salinity

Salinity in agricultural land is a major problem worldwide, placing a severe constraint on crop growth and productivity in many regions, and increased salinization of arable land is expected to have devastating global effects [18,38,39]. Though plants vary in their sensitivity to salt stress, high salinity causes water deficit and ion toxicity in many plant species.

Studies of transgenic plants demonstrated that compatible solutes, such as proline, trehalose and glycinebetaine, are accumulated in plants under salt stress at the millimolar range, playing an osmoprotective role in physiological responses and enabling the plants to better tolerate soil salinity [40,41]. Moreover, low levels of GB, applied exogenously or generated by transgenes for the biosynthesis of compatible solutes can induce the expression of certain stress-responsive genes, including those for enzymes that scavenge reactive oxygen species [40]. Furthermore, considerable efforts have therefore been made to investigate how genes respond to salt stress in various plants by using several approaches, including proteomics [21]. The effects of NaCl on the H_2_O_2_ content and CAT activity were studied in diverse groups of plants, such as a unicellular alga, *Chlorella* sp., an aquatic macrophyte, *Najas graminea*, and a mangrove plant, *Suaeda maritima*, all showing high tolerance to NaCl. Significant accumulation of H_2_O_2_ was observed in all the tested plants upon their exposure to high levels of NaCl, and CAT activity increased significantly in response to the NaCl treatment [42]. Interestingly, the same authors found that growing the plants in the presence of a high degree of salinity also resulted in the synthesis of new isoforms of CAT. In order to understand the role of some key genes in response to salt stress, Wang *et al.* [43] isolated a gene encoding a small GTPase (*MfARL1*), from a subtracted cDNA library in *Medicago falcate*. Transgenic seedlings constitutively expressing *MfARL1* had higher survival rates under salt stress. Salt stress led to a significant decrease in chlorophyll contents in wild-type plants, but not in transgenic plants. These accumulated less amounts of H_2_O_2_ and presented a lower oxidative damage than their wild-type counterparts when subjected salt stress, which can be mainly accounted for by the higher CAT activity. Interestingly, in tomato leaves and roots, peroxisomal CAT activity resulted to be higher in plants subjected to different degrees of salt stress compared to controls, whereas in other species, such as peas, CAT activity in purified leaf peroxisomes did not increase in response to salinity [44,45,46].

Another gene with a key role during salt and osmotic stress tolerance seems to be *AtWNK8* [47], mainly expressed in primary root, hypocotyl, stamen and pistil. Indeed, compared to the wild-type, the mutants overexpressing *wnk8* are more tolerant against severe salinity and osmotic stresses [47]. Under NaCl and sorbitol stresses, CAT activity in *wnk8* mutants is higher than in wild-type plants. The authors provided evidence that increased tolerance of *wnk8* mutants to salt stress is due to higher endogenous activities of CAT and GPX, together with higher proline synthesis and accumulation.

Some pretreatments on plants are recognized as valuable strategies to stimulate plant defenses against salt stress. For instance, Nounjan *et al.* [41] investigated the effect of other two exogenous osmoprotectants (proline and trehalose) on a salt-sensitive rice variety, where salt stress resulted in growth reduction, increase in the Na^+^/K^+^ ratio, increase in proline level and up-regulation of proline synthesis genes (pyrroline-5-carboxylatesynthetase, *P5CS*; pyrroline-5-carboxylate reductase, *P5CR*) as well as accumulation of H_2_O_2_, increasing of the activity of antioxidative enzymes, and up-regulation of genes encoding for antioxidant enzymes, as *CatC*. It seems that, although exogenous osmoprotectants did not alleviate growth inhibition during salt stress, they exhibited a pronounced beneficial effect during recovery period, showing higher percentage of growth recovery. The authors observed that the increase in CAT activity was most related to significant reduction in H_2_O_2_, particularly in the case of proline-treated plants. In tree species (various wild almond species), Sorkheh *et al.* [48] highlighted that the application of proline can alleviate the detrimental effects of abiotic stresses, such as salinity, allowing leaves to better face oxidative stress by acting as an efficacious H_2_O_2_ scavenger. Moreover, it was observed that salt stress induces significant changes in CAT activities in various wild almond species [49]. In another work, Gondim *et al.* [50] evaluated the effects of H_2_O_2_ leaf spraying pretreatment on plant growth. The experiment revealed that H_2_O_2_ spraying increased antioxidant enzyme activities, and that CAT was the most responsive of these enzymes to H_2_O_2_. Increased CAT activity appears linked to gene expression regulation, and lower oxidative damage was detected in plants with higher CAT activity, considering the protective function of this enzyme.

## 3. Ascorbate Peroxidase (APX)

### 3.1. Drought and Salinity

**Figure 3:**
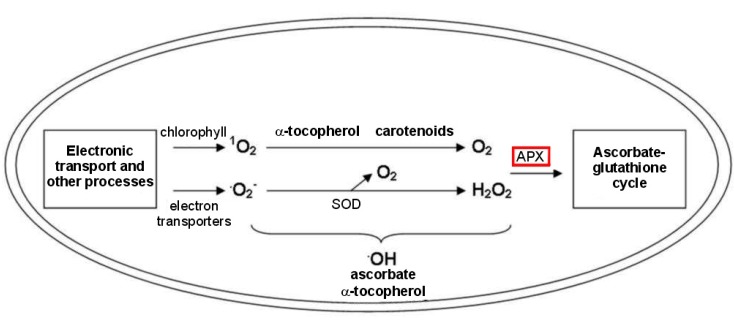
Production and scavenging of ROS in chloroplasts of plants subjected to abiotic stresses, showing the key role of ascorbate peroxidases (APX) in ROS scavenging patterns.

A better knowledge of the effects of water deficit and salt excess on plant biochemistry has a primary importance for improved management practices, breeding programmes and for predicting plant growth and product quality. In this regard, a major hydrogen peroxide detoxifying system in plant cells under abiotic stressors is the ascorbate-glutathione cycle, in which ascorbate peroxidase (APX) isoenzymes play a key role in catalyzing the conversion of H_2_O_2_ into H_2_O, using ascorbate as a specific electron donor [51,52], particularly in the chloroplast (Figure 3).

Mutant plants *APX* genes showed alterations in growth, physiology and antioxidant metabolism revealing those enzymes involvement in the normal plant development. The expression of *APX* genes is strictly regulated in response to drought and salt stresses as well as during plant development [23,53]. The genes encoding APXs are particularly important in maintaining the homeostasis of ascorbate (AsA) and glutathione (GSH), two non-enzymatic antioxidants within the context of cellular redox homeostasis and redox signaling, and directly or indirectly involved in maintaining high photosynthetic rates in plants under adverse environmental conditions [23,34]. For instance, in saline soils and/or when drought limits the CO_2_ fixation, the excess excitation energy is dissipated in the light harvesting antennae as heat by zeaxanthin, that is formed by successive de-epoxidation of the xanthophyll cycle pigments violaxanthin and antheroxanthin. The deepoxidase, which is bound to the lumen side of the thylakoid membrane, is dependent on AsA as a cofactor [52]. Etelib *et al.* [54] found that transgenic tobacco plants accumulating greater amounts of AsA have an enhanced tolerance to salt stress. In addition, AsA is involved in other functions such as plant growth, gene regulation, modulation of some enzymes, and redox regulation of membrane-bound antioxidant compounds in plants under both drought and salt stress [51].

In order to identify and study factors involved in stress responses, by using mutants, it was found that particular small RNA molecules, are involved. These elements of RNA metabolism participate in the regulation of different pathways linked to environmental stress conditions, including drought and salinity [55]. In particular, in the context of antioxidant defence, the role of these small regulatory non-coding microRNAs (miRNAs) has been recently established, also in the regulation of antioxidant enzymes during the plant response to drought and salinity stresses. For example, some miRNAs identified in cotton plants (miR156-SPL2, miR162-DCL1, miR159-TCP3, miR395-APS1, and miR396-GRF1) and their predicted targets were found to be differentially expressed under dose- and tissue-dependent salinity and drought [56,57]. Very recently, in the same plant, others miRNAs were identified, of which at least 18 and 27 are salt specific and drought specific, respectively [58]. Salinity and drought stresses were able to up-regulate and down-regulate, respectively, miRNAs expression, miR395 being the most sensitive to both stress levels, also in French bean seedlings [59]. In addition, in the same experiments, the expression of *APX*-coding gene was also found to be up-regulated by salinity and drought stresses, indicating a pivotal role of molecular regulation mechanisms induced by these kind of abiotic stresses.

### 3.2. Drought

**Figure 4:**
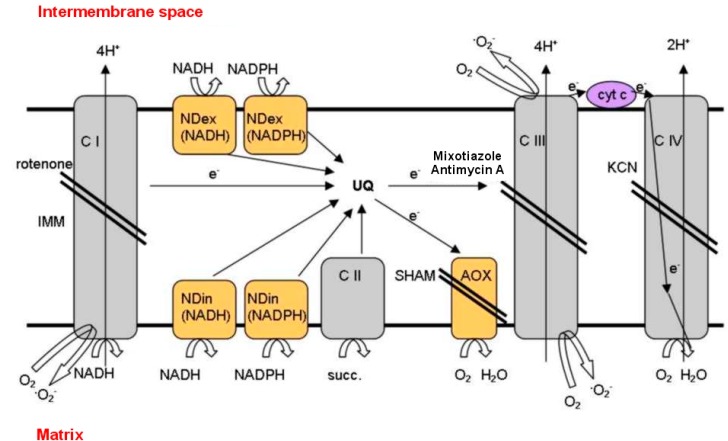
Electron transport chain of the inner mitochondrial membrane of plant cells. Enzymes in yellow and membrane-bound complexes in grey; inhibitors of electron transport are flanked by a double bar. C I = complex I, NADH dehydrogenase; C II = succinate dehydrogenase; C III = ubiquinol-cytochrome bc1 reductase; C IV = cytochrome c oxidase; cyt c = cytochrome c; SHAM = salicylichydroxamic acid.

The redox state of the chloroplast and mitochondria is maintained by a delicate balance between energy production and consumption, and affected by the need to avoid increased production of ROS. These demands are especially critical during exposure to extreme environmental conditions, such as drought alone or in combination with other environmental stresses [60,61]. Under water deficit conditions, ROS and redox cues, generated mainly during the mitochondrial electron transport (Figure 4), are essential for maintaining normal energy and metabolic fluxes, optimizing different cell functions, activating acclimation responses through retrograde signalling, and controlling whole-plant systemic signalling pathways [62]. In the complexity of the regulation network of plant antioxidant defences, APX is an antioxidant enzyme that plays a key role in drought stress responses and following recovery from drought [28,63,64]. For instance, Kausar *et al.* [65] observed a clear increase in APX amount and activity with increasing drought in soybeans, detected by western blotting, enzyme activity assay and biophoton emission techniques. In another piece of work, Zarei *et al.* [66] measured APX content in transgenic tobacco (*Nicotiana tabacum*, cv. Wisconsin), over expressing a Δ-1-pyrroline-5-carboxylate synthase (P5CS) gene, and non-transgenic plants as control. Drought stress was applied using polyethylene glycol (PEG) 6000 at different concentrations. The authors observed that APX activity increased under drought stress and the highest activity was observed in 10% and 20% of the PEG treatment, suggesting that P5CS is an inducible gene and that the induction of APX activity is involved in drought tolerance mechanism. Research on the relationships between drought and APX in tree species are very scarce. For example, in a wild species of almond (*Prunus* spp.), after 60 days of water, the activity of APX and other enzymes of the ascorbate-glutathione cycle increased in relation to the severity of drought stress, whereas they recovered during the following rewatering phase [67]. The hypothesis that application of exogenous glycine betaine may attenuate the effects of mild water deficit in leaf gas exchange and lipid peroxidation in *Carapa guianensis* was examined by Cruz *et al.* [68]. They found that the application of 25 and 50 mM glycine betaine caused significant increases in APX activity in drought-stressed plants. Thus, glycine betaine attenuated lipid peroxidation in drought-stressed plants through positive modulation of APX activity.

In order to understand the role of cytosolic antioxidant enzymes in drought stress protection, transgenic tobacco (*Nicotiana tabacum* cv. *Xanthi*) plants overexpressing cytosolic APX (cyt*apx*) were produced and tested for tolerance against mild water stress [28]. Their results showed that the simultaneous overexpression of *apx* gene in the cytosol of transgenic tobacco plants alleviates, to some extent, the damage produced by water stress conditions. This was correlated with higher water use efficiency and better photosynthetic rates. In general, oxidative stress parameters, such as lipid peroxidation, electrolyte leakage, and H_2_O_2_ levels, were higher in non-transformed plants than in transgenic lines, suggesting that, at the least, overexpression of cyt*apx* (cytosolic APX) protects tobacco membranes from water deficit. In recent work, Zhang *et al.* [69] studied the function of rice *OsAPX2* gene using a T-DNA knockout mutant under the treatment of drought stress, founding that loss of function in *OsAPX2* affected the growth and development of rice seedlings, resulting in semi-dwarf seedlings, yellow-green leaves, leaf lesion mimic and seed sterility. *OsAPX2* mutants had lower APX activity and were sensitive to drought, whereas overexpression of *OsAPX2* increased APX activity and enhanced stress tolerance. The important role of APX during plant responses to drought has been recently depicted by Singh *et al.* [70]. In their work, a full-length *SbpAPX* cDNA, encoding peroxisomal ascorbate peroxidase, was cloned from *Salicornia brachiata* Roxb., an extreme halophyte. The deduced amino acid sequence of the *SbpAPX* gene showed characteristic peroxisomal targeting sequences and a C-terminal hydrophobic region of 39 amino acid residues containing a transmembrane domain of 23 amino acid residues. Transgenic plants over-expressing *SbpAPX* gene showed enhanced drought stress tolerance compared to wild-type plants.

### 3.3. Salinity

Ascorbate peroxidase is one of the major members of the ROS scavenging system that plays an important role in improving saline-alkali tolerance in plants, detoxifying H_2_O_2_ in different cell compartments, being involved in the homeostasis of AsA, and balancing the intracellular ROS messenger network [71,72,73]. A series of studies have demonstrated that mutants deficient in cytosolic ascorbate peroxidases are susceptible to the oxidative damage induced by salinity ([74,75,76,77] and references within). To fortify the antioxidant capacity of plum plants, Diaz-Vivancos *et al.* [71] produced transgenic plum plants expressing the cyt*apx* (cytosolic APX) genes under the control of the *CaMV35S* promoter. In *in vitro* plum plants against salt stress (100 mm NaCl), transgenic seedlings expressing cyt*apx* showed an enhanced tolerance to salt stress and also exhibited higher contents of the non-enzymatic antioxidants AsA and GSH than non-transformed controls. Recently, Guan *et al.* [78] investigated the relationship between the APX (*PutAPX*) gene of *Puccinellia tenuiflora* as a perennial wild grass able to grow in extreme saline-alkali soil environments. Interestingly, the overexpression of *PutAPX* in trasgenic *Arabidopsis thaliana* significantly increased the tolerance of plants treated with 150 and 175 mM NaCl and decreased the extent of lipid peroxidation. The transgenic seedlings presented higher chlorophyll content and lower H_2_O_2_ content than that of wild-type plants under both normal conditions and 200 mM NaCl stress. Moreover, the expression of APX proteins and enzyme activity in the transgenic seedlings increased to levels that were greater than two-fold higher than those found in wild-type plants exposed to 200 mM NaCl. On the other hand, plants silenced for APXs can be able to up-regulate other peroxidases, making the mutants able to cope with salt stress, similar to non-transformed plants. Indeed, Bonifacio *et al.* [74] demonstrated that rice APx1/2s mutants (double mutants for cytosolic APXs) exhibited an altered redox homeostasis, as indicated by increased levels of H_2_O_2_ and AsA and GSH redox states, but the antioxidative compensatory mechanism displayed by the mutants was associated with increased expression of *OsGpx* genes, which resulted in higher GPX activity in the cytosolic and chloroplastic fractions. Therefore, it seems that the deficiency in cytosolic APXs can be effectively compensated by the up-regulation of other peroxidases. As in the case of drought stress, hormonal balance appears to be involved in APX regulation. Salicylic acid (SA) is known to affect photosynthesis under normal conditions and induces tolerance in plants to biotic and abiotic stresses through influencing physiological processes. In a study of Nazar *et al.* [79], physiological processes were compared in cultivar of mungbean with a different degree of tolerance against salinity. The authors examined how much these processes were induced by SA treatment to alleviate decrease in photosynthesis under salt stress. Applications of 0.5 mM SA increased nitrogen and sulfur assimilation, GSH content and activity of APX and glutathione reductase. This resulted in the increase in photosynthesis under non-saline conditions, alleviated the decrease in photosynthesis under salt stress, and also helped in restricting Na^+^ and Cl^−^ content in leaf and maintaining higher efficiency of PSII, photosynthetic N-use efficiency, and water relations.

## 4. Conclusions and Perspectives

For a long time, H_2_O_2_ has been considered not only a simple by-product of oxidative stress in plants but also a molecule involved in the regulation of gene expression during the exposure of plants to biotic and abiotic stresses [9,80,81].

A study conducted by using DNA microarray technology demonstrated that cells of *Arabidopsis* exposed to H_2_O_2_ changed the expression levels of 175 genes, and 113 genes were induced to encode for proteins acting as antioxidants, or as stress defense responses, or as signaling proteins [82]. After some years, Luo *et al.* [83] used the same microarray-based screening technology for demonstrating that 42 genes of peanut plants were up-regulated as a response to both biotic and abiotic stresses (consisting in *Aspergillus parasiticus* infection and drought, respectively), when they were imposed together, and 52 genes were up-regulated by drought stress alone. Nowadays, it is widely recognized that H_2_O_2_ plays a role in the activation of the genes involved in the acclimation, stress tolerance, and other defense responses ([2,77] and references within).

**Figure 5:**
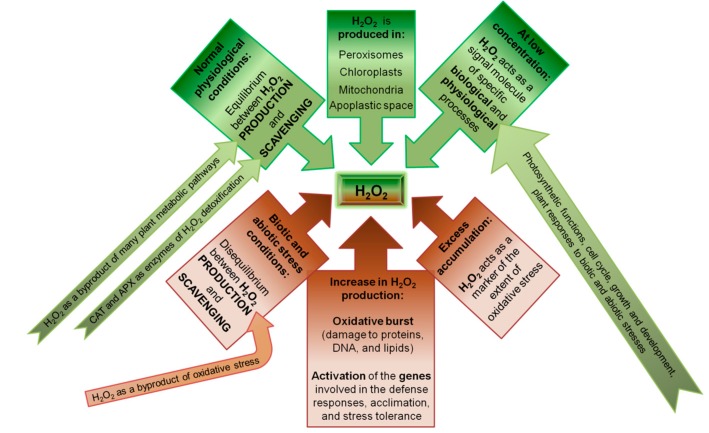
Role of the H_2_O_2_ under normal physiological conditions (above, indicated with green color), and under stress conditions (below, indicated with brown color).

The studies discussed show that H_2_O_2_ is a ubiquitous metabolite in plants and that it has many diverse and important functions in H_2_O_2_-mediated signal transduction cascades and gene regulation, as schematized in Figure 5. The intra-cellular distribution and functions of H_2_O_2_ are still not completely clear, and more experimentation is required to determine whether the chloroplasts, mitochondria, peroxisomes, and cytosol H_2_O_2_ pools fulfill different roles and how they are inter-connected. In the immediate future, studies are required to elucidate how the function of the different H_2_O_2_ pools are coordinated by APX and CAT and whether or not they have additional functions in plants.
